# Supplementary material for: The human cytomegalovirus vGPCR UL33 is essential for efficient lytic replication in epithelial cells
Source: J Virol. 2026 Aug 31;100(9):e00664-26. doi: 10.1128/jvi.00664-26 (PMC13595971; doi:10.1128/jvi.00664-26)
Supplement: Supplementary file 1 [file jvi.00664-26-s0001.pdf]

Freeman, Dooley, et. al. Supplemental Table 1

	Exon 1 (TB40/E coordinates)	Exon 2 (TB40/E coordinates)	Exon 3 (TB40/E coordinates)	Exon 4 (TB40/E coordinates)	Description	Read count supporting this	
UL33 Genbank Annotation			43847-43870	43992-45206	Contains E3,E4		
6hr	41716-41814		43793-43870	43871-45426	Contains E1,E3,E4*	330	
**6hr**	**41716-41814**		**43793-43870**	**43992-45426**	**Contains E1,E3,E4**	**465**	**#**
**6hr**		**43435-43494**	**43793-43870**	**43992-45426**	**Contains E2,E3,E4**	**1480**	**#**
6hr			43672-43870	43992-45426	Contains E3,E4	2885	
6hr		43435-43494	43793-43870	43871-45246	Contains E2,E3,E4*	406	
24hr	41747-41814		43793-43870	43871-44835	Contains E1,E3,E4*	1245	
24hr		43441-43494	43793-43870	43871-44835	Contains E2,E3,E4*	11489	
24hr		43462-43494	43642-43870	43871-44835	Contains E2,E3,E4*	3794	
48hr	41777-41814			43992-45206	Contains E1,E4	27366	
48hr			43653-43870	43871-44837	Contains E3,E4*	16134	
72hr	41712-41814		43793-43870	43871-45012	Contains E1,E3,E4*	11813	
72hr			43646-43870	43871-45012	Contains E3,E4*	54531	
**72hr**			**43646-43870**	**43992-45206**	**Contains E3,E4**	**15925**	**#**

***** Indicates RNAs contain E3 and E4 as well as the intervening intron between E3 and E4
# Indicates coordinates used for the basis of UL33 expression constructs described in Figures 10 & 12

Supplemental Table 1. Read Counts from RNAseq analysis indicating UL33 transcription containing additional upstream exons.

Splice junction	Forward (5' to 3') [coordinates]	Reverse (5' to 3') [coordinates]	Product size (bp)
Exon 1 to Exon 3(5' short)	GTGGAAGTCGTAGGCATCC [41777-41795]	CGAGTTGTGGATGATGGTGTC [43850-43870]	116
Exon 1 to Exon 4	GTGGAAGTCGTAGGCATCC [41777-41795]	TGAATGTGTTGAGTACGGCT [44069-44088]	135
Exon 2 to Exon 3(5' long)	GCGCCTTACCACGATCAC [43470-43487]	CAACTTTCTGCGCCATCTCA [43707-43726]	106
Exon 2 to Exon 3(5' short)	GCGCCTTACCACGATCAC [43470-43487]	CGAGTTGTGGATGATGGTGTC [43850-43870]	103
Exon 2 to Exon 4	GCGCCTTACCACGATCAC [43470-43487]	TGAATGTGTTGAGTACGGCT [44069-44088]	122
Exon 3 to Intron 3_4	GACACCATCATCCACAACTCG [43850-43870]	GCGGCGCTTAAAAGAAAACC [43926-43945]	96
Exon 3 to Exon 4	GACACCATCATCCACAACTCG [43850-43870]	TGAATGTGTTGAGTACGGCT [44069-44088]	118
Intron 3_4 to Exon 4	GGTTTTCTTTTAAGCGCCGC [43926-43945]	CATTGATGTGCGGAGGAGTG [44003-44022]	97

Supplemental Table 2. Oligonucelotide primer pairs used to confirm presence of UL33 transcript splice junctions during HCMV infection of NuFF-1 and ARPE-19 cells.

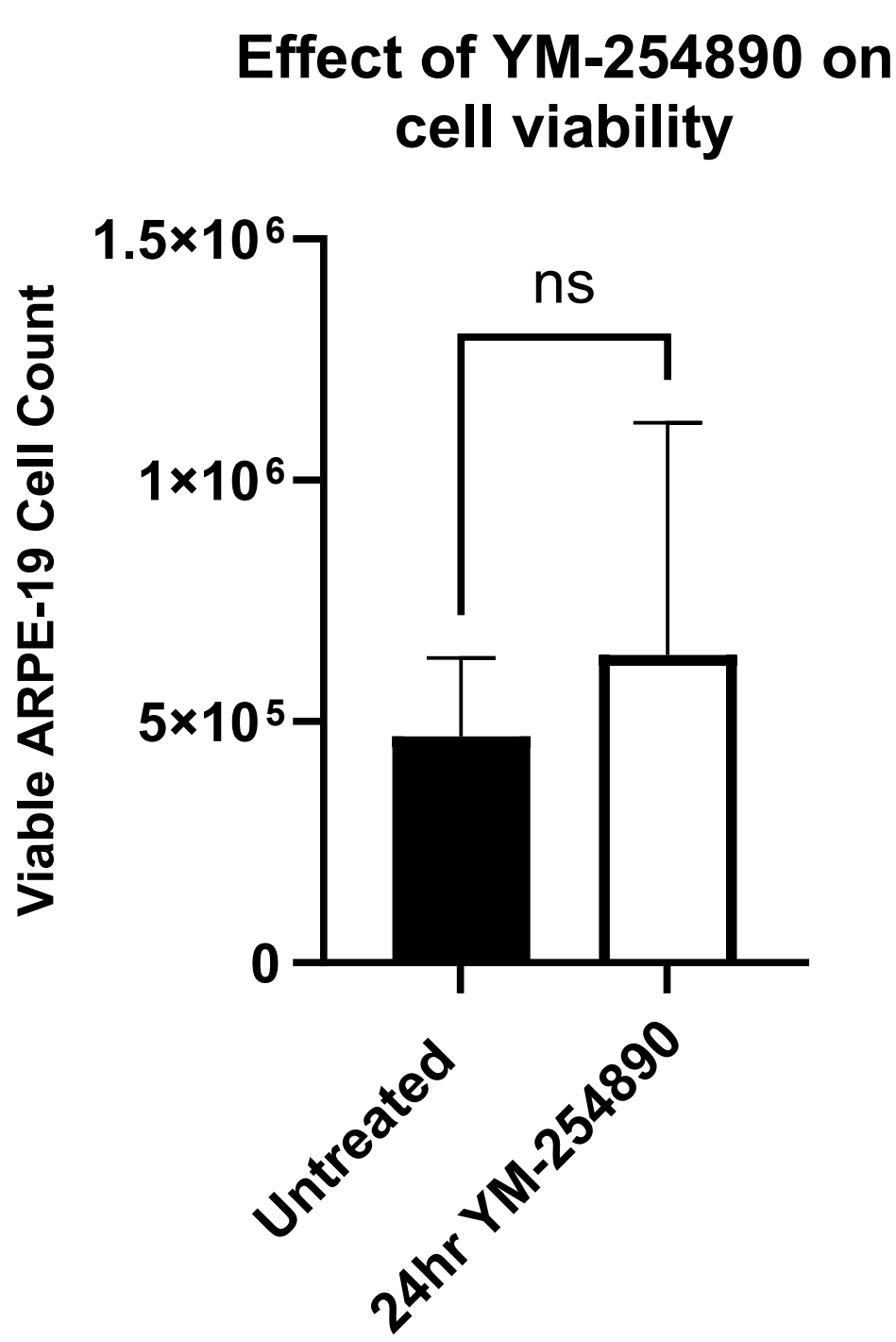

Supplemental Figure 1. YM-254890 does not impact cell viability. ARPE-19 cells were treated with 100nM for 24 hours to match the treatment duration for IP accumulation assays performed in Figure 8. Viable cells in untreated or treated wells were enumerated using a hemacytometer. Error is shown as standard deviation from three independent experiments, each performed in triplicate. Pairwise t-tests between the induced and uninduced groups were used to determine statistical significance; $*p < 0.05$, $**p < 0.01$.
